# Could Mean Platelet Volume Be a Reliable Indicator for Acute Mesenteric Ischemia Diagnosis? A Case-Control Study

**DOI:** 10.1155/2016/9810280

**Published:** 2016-10-11

**Authors:** Vermi Degerli, Isil Ergin, Fulya Yilmaz Duran, Mehmet Akif Ustuner, Ozgur Duran

**Affiliations:** ^1^Department of Emergency Medicine, Izmir Bozyaka Training and Research Hospital, Bozyaka, Izmir, Turkey; ^2^Department of Public Health, Ege University School of Medicine, Bornova, Izmir, Turkey; ^3^Department of Anaesthesiology and Reanimation, Izmir Bozyaka Training and Research Hospital, Bozyaka, Izmir, Turkey; ^4^Department of Surgery, Dr. Abdurrahman Yurtaslan Ankara Oncology Training and Research Hospital, Yenimahalle, 06200 Ankara, Turkey

## Abstract

*Objective*. Acute mesenteric ischemia (AMI) is a disease, usually seen in elderly people and accompanied by comorbid diseases. Mean platelet volume (MPV), the significant indicator of platelet activation and function, is associated with AMI. In this study, we considered that we can use MPV as a reliable indicator in the diagnosis of AMI.* Methods*. This study was conducted among AMI patients with two control groups. Age, gender, MPV, platelet count, concomitant diseases, abdominal computed tomography, and patient outcomes were recorded for evaluation. Control group I contained 41 healthy patients whose ages-genders were matched. Control group II contained 41 patients with no AMI, whose ages-genders-concomitant diseases were matched.* Results*. Of the total 41 AMI patients, 22 were female and 19 were male. The average age of them was 72.12 ± 13.2 (44–91) years. MPV was significantly increased in the AMI (*p* = 0.001) and control group II (*p* < 0.001) in comparison with healthy control groups. In the comparison of the AMI patients with their matched controls for concomitant diseases, no statistical difference was found in the MPV values.* Conclusion*. MPV may be used as an indicator of AMI only if the patient has no concomitant diseases. The existence of a concomitant disease brings into question the reliability of high MPV values as a suitable indicator.

## 1. Introduction

Acute mesenteric ischemia (AMI) is a rare disease, and it accounts for approximately 0.1% of patients getting treatment in emergency department [1–3]. The causes of AMI are embolism (50%), thrombosis (20%), nonocclusive factors (20%), and venous thrombosis (10%). The most common risk factors are age, myocardial infarction, dysrhythmias (especially atrial fibrillation), atherosclerosis, cardiac failure, hypercoagulability, intra-abdominal trauma or infection, and malignancy [1, 2, 4]. Early diagnosis and surgical intervention are very important for reducing mortality. Yet, the AMI diagnosis is difficult, because clinical signs are nonspecific, and there is no exact laboratory marker for AMI. Gold standard for the diagnosis of AMI is multidetect row computed tomography. Serum plasma markers such as leukocytes, D-dimer, ischemia-modified albumin, urinary and plasma fatty acid-binding proteins, and serum lactate levels do not have sufficient diagnostic accuracy for the diagnosis of AMI [1, 3].

Platelet aggregation and coagulation activation are fundamental for clot formation in the arterial and venous thrombosis [5, 6]. AMI is seen in both arterial and venous system; the origin is 80% thromboembolic disease. Due to an increase in thrombogenic activity, an increase in MPV is to be expected. Platelet volume is a marker of platelet function and activation that is readily measured as MPV. It can be routinely measured by automated cell counter. It is a simple parameter inexpensive to obtain and easy to interpret. Circulating platelets may differ according to their density, reactivity, and size. The larger platelets contain more dense granules. They are metabolically and enzymatically more active than smaller ones. They have more thrombotic and inflammatory property [5–12].

There is only a few studies evaluating the role of MPV in AMI patients [12–14]. Our research questions are as follows: (i) Does MPV increase in AMI patients in comparison with healthy controls? (ii) Does MPV increase in patients with concomitant diseases in comparison with healthy controls? (iii) Can the MPV increase be attributed to AMI only?

## 2. Materials and Methods

This study was conducted at emergency department of our hospital between January 2008 and December 2014. After ethical committee approval was obtained, all baseline data were collected retrospectively from patients' medical records. The data consisted of demographics, concomitant diseases, laboratory tests including complete blood count, imaging tests including abdominal computed tomography, and patient outcomes. Patients with history of malignancy, chronic hematological diseases, autoimmune disease, hepatic dysfunction, acute or chronic inflammatory disease, pregnancy, and cranial trauma were excluded. The outcomes of the cases were grouped as survival or exitus from the records.

Regarding effect size as 0.55 and *α* error probability 0.05, the power of this study is 80% for 41 cases and 41 controls.

The case group of the study was comprised of AMI patients. Hospital records of 41 patients (age ≥ 18 years) who were operated on with the diagnosis of AMI and pathological specimens revealed AMI were reviewed retrospectively. The study included two control groups: control group I: age-sex-matched 41 healthy patients with no concomitant diseases; control group II: age-sex-concomitant disease-matched 41 patients who were not diagnosed for mesenteric ischemia.

During admission to the emergency department, patients peripheral venous blood samples were drawn by careful vein puncture in 20 G sterile syringe. For measurement of MPV and platelet count (PC), 2 mL of blood was drawn into a vacutainer tube, containing EDTA as an anticoagulant and analyzed within 60 minutes in an automated blood cell counter (BC-6800 analyzer, Mindray, China). Normal range for MPV was 7–10.7 femtoliter (fL) in our center laboratory.

### 2.1. Statistical Analysis

Statistical analysis was performed using SPSS 20.0 for Windows (SPSS, Chicago, IL, USA). The results were reported as mean ± standard deviation (SD) for the quantitative variables and percentages for the categorical variables. The groups with the different MPV counts and PC were compared by using Student's *t*-test and its nonparametric variant Mann-Whitney *U* (MWU) as appropriate. The Pearson test was used for correlation of MPV and PCs. Statistical significance was defined as *p* values less than 0.05.

Comparisons were made for (1) AMI and control group I, (2) AMI and control group II, and (3) control group I and control group II.

In the receiver operating characteristic (ROC) curve analysis, only the control group I has been compared with AMI group. This comparison has been chosen because the comparison between all non-AMI patients and AMI patients resulted in a ROC curve with area under the curve = 0.575 (SE = 0.055, *p* = 0.176), while the comparison between control group I and AMI resulted in a better ROC curve with area under the curve = 0.690 (SE = 0.059, *p* = 0.003). To calculate the best cut-off in ROC curve, Youden's index was used in which “sensitivity + specificity − 1” was maximal.

## 3. Results

Among the patients with AMI, there were 22 females (53.7%) and 19 males (46.3%); the average age of them was 72.12 ± 13.2 (44–91) years. All patients had a history of concomitant disease; and the four diseases following were hypertension, cardiovascular disorders, diabetes mellitus, and atrial fibrillation, consecutively. Baseline characteristics for AMI are summarized in Table 1. Control I and control II groups' average ages were 72.34 ± 13.0 (45–91) and 72.21 ± 13.4 (41–90) years, respectively.

Table 2 shows the laboratory characteristics of case and control groups. While PC does not show any significant difference in the comparison of three groups, MPV is significantly increased in the ischemia (*p* = 0.001) and concomitant disease group (*p* < 0.001) in comparison with the healthy group. However, no significant difference was found between the ischemia and the concomitant disease group (*p* = 0.563).

In Table 3, the results of the MPV comparisons for concomitant disease groups with (cases) and without (control group II) AMI are presented. In the comparison of the AMI patients with their matched controls for concomitant diseases, no statistical difference was founded in the MPV values (Table 3, *p* > 0.05).

In the comparison of the outcomes (survival/exitus) of mesenteric ischemia, there was no significant difference in the MPV (MWU = 193.5, *p* = 0.956) and PC (MWU = 134.5, *p* = 0.095). In the AMI patients, control I and control II groups, there was seen a low level of negative correlation in PC and MPV, significantly. The Pearson correlation coefficients in each group were 0.364, 0.361, and 0.334, respectively (*p* < 0.05).

**Figure 1:**
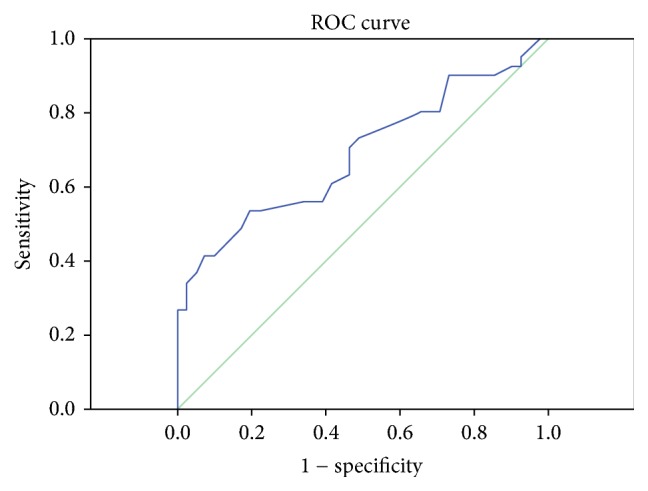
Receiver operating characteristic curve results for mean platelet volume in patients with acute mesenteric ischemia.

ROC curve analysis suggested that the best MPV value cut-off point for AMI was 8.6 fL, sensitivity was 70%, and specificity was 53% (area under the curve = 0.690) (Figure 1). Positive likelihood ratio was 1.52 and negative likelihood ratio was 1.83 for this cut-off.

## 4. Discussion

This study is a first attempt to evaluate the MPV value among AMI patients in comparison with two controls: healthy and concomitant disease with no AMI. An important finding is obtained from this study that reliance on high MPV values in diagnosis of AMI may be insufficient in the existence of concomitant diseases.

When we examine the literature, Altintoprak et al. are the first to investigate the relationship between MPV and AMI. In their study they reported that MPV values were higher in nonsurvivors (*n* = 15, MPV = 9.01) than in survivors (*n* = 15, MPV = 7.8) [14]. Bilgiç et al. investigated whether MPV was associated with outcome of AMI and reported that MPV values were higher in nonsurvivors (*n* = 35, MPV = 8.4 fL) than in survivors (*n* = 26, MPV = 7.6 fL) [12]. In our study, it is revealed that MPV was significantly higher in patients with AMI compared to healthy controls (9.6 fL and 8.7 fL, resp.). In a recent study, Türkoğlu et al. reported that MPV was higher in patients with AMI compared to healthy volunteers (9.4 fL and 7.4 fL, resp.) [13]. MPV is a simple parameter that indicates the platelet size. When compared with small ones, large platelets are more prone to aggregation and inflammation, and they produce prothrombotic substances. In addition, large platelets are less sensitive to the inhibitory effect of prostacyclin on aggregation and secretion compared to small ones [5]. Therefore, higher MPV values in patients with AMI can be explained by inflammatory and thrombotic features of the disease.

To our knowledge, there is no study in the literature that evaluates if higher MPV values in patients with AMI arise from ischemia or concomitant diseases of these patients. Previous studies have reported that there is increase in MPV in patients with coronary artery disease [7, 11, 15, 16], hypertension [8, 9, 17], diabetes mellitus [11, 18], atrial fibrillation [11], congestive heart failure [7], acute ischemic brain stroke [10, 11, 19–21], and venous thromboembolism [6, 22]. However, some authors have revealed contrasting data as well [23–26].

According to our study results, when compared with healthy controls, MPV was significantly higher in patients without diagnosis of AMI but with concomitant diseases (control group II). Regarding this finding, the MPV level of the patients with AMI may be confounded by their concomitant diseases. In the study herein, most of the AMI patients were geriatric, and they had concomitant diseases. So, we presume that MPV might be higher in these patients before the diagnosis of AMI. The elevated MPV in AMI, when adjusted for concomitant diseases, appears related to concomitant diseases rather than AMI.

We also reported that MPV value and PC are not associated with the mortality of the AMI patients. Altintoprak et al. and Bilgiç et al. disclosed that there are higher MPV values in the exitus group compared with the survival group in patients with AMI [12, 14]. They revealed that higher MPV values are poor prognostic parameters among patients with AMI. Our study results do not show higher MPV associated with mortality. This can be explained with the high age profile of our AMI patient group compared with the studies revealed.

We determine a negative correlation between MPV values and PC parallel to findings of previous studies. Although the relationship between MPV and PC is not fully understood, some studies reported that increase in volumes of platelets is associated with decrease in platelet count. Under normal conditions, the circulating platelet mass (platelet count × MPV) is kept constant [5, 6, 9, 11].

In our study the cut-off value for MPV was determined to be 8.6 fL with 70% sensitivity and 53% specificity. While Bilgiç et al. reported MPV level cut-off points for AMI 8.1 fL with 60% sensitivity and 73% specificity, Türkoğlu et al. reported MPV level cut-off points for AMI 8.1 fL with 83.2% sensitivity and 80% specificity [12, 13]. Patients diagnosed with AMI are geriatric and often have concomitant diseases. MPV values are reported higher especially in cardiovascular-related comorbid diseases [6–11, 15–22]. According to our study results, higher MPV values were reported in patients with diagnosis of AMI who have concomitant diseases and in patients without diagnosis of AMI but with concomitant diseases (control group II). Thus we can explain high MPV values not only by AMI but also with concomitant diseases. Regarding this finding, we can explain lower sensitivity and specificity values in our study.

There are some limitations in this study. We neglected the drugs used by the patients in our study group. In a few studies, it is found that antiplatelet drugs and lipid-lowering drugs have effects on platelet size; but the results are controversial [5, 7, 20]. The study is a retrospective evaluation limited with information in the patient data

## 5. Conclusions

MPV may be used as an indicator of AMI only if the patient has no concomitant diseases. The existence of a concomitant disease brings questions about the reliance of high MPV values as a good indicator in diagnosis of AMI. Executing investigations in a prospective study designed with larger patient groups may bring more insight to the current findings.

## Figures and Tables

**Table 1:** Demographic and clinical characteristics of the mesenteric ischemia patients.

Characteristics	Mesenteric ischemia group (*n* = 41)

Age (years ± SD)	72.12 ± 13.2
Female, *n* (%)	22 (53.7)
Computed abdomen tomography positive, *n* (%)	25 (86.2)^*∗*^
Outcome patient/exitus, *n* (%)	23 (57.5)^*∗∗*^
Hypertension, *n* (%)	25 (61.0)
Coronary artery accident, *n* (%)	20 (48.8)
Diabetes mellitus, *n* (%)	14 (34.1)
Atrial fibrillation, *n* (%)	9 (22.0)
Heart failure, *n* (%)	5 (12.2)
Cerebrovascular disease, *n* (%)	5 (12.2)
Chronic renal failure, *n* (%)	4 (9.8)
Acute renal failure, *n* (%)	3 (7.3)
Chronic obstructive pulmonary disease, *n* (%)	3 (7.3)
Epilepsy, *n* (%)	1 (2.4)
Deep vein thrombosis, *n* (%)	1 (2.4)

^*∗*^Among 29 patients who had CT.

^*∗∗*^Among 40 patients treated.

**Table 2:** Laboratory characteristics of AMI group and control groups.

Variables	AMI group (*n* = 41)	Control group I^*∗*^ (*n* = 41)	*p*	Control group II^*∗∗*^ (*n* = 41)	*p*

PC (×10^3^/mL)	232.05 ± 86.26	252.51 ± 60.10	0.216	264.85 ± 73.81	0.068
MPV (fL)	9.65 ± 1.31	8.79 ± 0.80	0.001	9.83 ± 1.47	0.563

AMI, acute mesenteric ischemia; PC, platelet count; MPV, mean platelet volume.

^*∗*^Age- and sex-matched from healthy 41 patients.

^*∗∗*^Age, sex, and concomitant disease-matched from 41 patients, but no mesenteric ischemia.

**Table 3:** The results of the MPV comparisons of concomitant disease groups with (cases) and without (control group II) AMI.

Concomitant disease	AMI group (*n* = 41)	Control group II (*n* = 41)	MPV MWU^**∗**^	MPV *p*

Hypertension, *n*	25	26	305.000	0.706
Coronary artery accident, *n*	20	20	159.000	0.267
Diabetes mellitus, *n*	14	14	71.000	0.214
Atrial fibrillation, *n*	9	8	23.000	0.210
Heart failure, *n*	5	4	4.000	0.142
Cerebrovascular disease, *n*	5	5	6.500	0.209
Chronic renal failure, *n*	4	2	4000	1.000
Acute renal failure, *n*	3	1	0.0	0.180
Chronic obstructive pulmonary disease, *n*	3	3	0.0	0.100
Epilepsy, *n*	1	1	0.0	0.317
Deep vein thrombosis, *n*	1	1	0.0	0.317

^*∗*^Mann-Whitney *U*.
